# The Ratio of Tibial Slope and Meniscal Bone Angle for the Prediction of ACL Reconstruction Failure Risk

**DOI:** 10.1055/s-0038-1668111

**Published:** 2018-08-20

**Authors:** Steffen Sauer, Robert English, Mark Clatworthy

**Affiliations:** 1 Department of Sports Traumatology, Aarhus University Hospital, Aarhus, Denmark; 2 Department of Orthopaedic Surgery, Middlemore Hospital, The University of Auckland, Auckland, New Zealand

**Keywords:** ACL, tibial slope, LTPS, MBA, FMIA, meniscal bone angle

## Abstract

**Background** A growing body of research is indicating that the tibial slope and the geometry of the tibiofemoral meniscal–cartilage interface may affect the risk of anterior cruciate ligament reconstruction (ACLR) failure. Increased lateral tibial posterior slope (LTPS) and reduced meniscal bone angle (MBA) are associated with increased risk of anterior cruciate ligament (ACL) injury. The significance of a LTPS–MBA ratio regarding the prediction of ACL failure risk remains unknown. As LTPS and MBA may eventually potentiate or neutralize each other, it is expected that a low LTPS–MBA ratio is associated with high chance of ACL graft survival while a high LTPS–MBA ratio is associated with high risk of ACL failure.

**Material and Methods** Out of 1,487 consecutive patients who underwent hamstring ACLR between August 2000 and May 2013, 54 ACLR failures with intact lateral menisci were included in this study and matched one-to-one with 54 control participants by age, sex, graft, surgical technique, and graft fixation method. Control participants had undergone ACLR without signs of lateral meniscal injury, graft failure, or insufficiency. MBA and LTPS were assessed on magnetic resonance imaging. Logistic regression was used to identify LTPS/MBA key cut-off ratios.

**Results** In this cohort, a LTPS–MBA ratio under 0.27 was associated with a 28% risk of ACLR failure (36% of patients), while a ratio exceeding 0.42 was associated with an 82% risk of ACLR failure (31% of patients). The odds of ACL failure increased by 22.3% per reduction of 1 degree in MBA (odds ratio [OR], 1.22; 95% limits, 1.1–1.34). No significant association was found between LTPS and the risk of ACL graft failure in transtibial ACLR, while the odds of ACL failure increased by 34.9% per degree of increasing LTPS in transportal ACLR (OR, 1.34; 95% limits, 1.01–1.79). No significant correlation was found between MBA and LTPS ( *p* = 0.5).

**Conclusion** Reduced MBA was associated with significantly increased risk of ACL graft failure. A ratio of LTPS and MBA was found to be useful for the prediction of ACLR failure risk and may preoperatively help to identify patients at high risk of ACLR failure. This may have implications for patient counseling and the indication of additional extra-articular stabilizing procedures.

The anterior cruciate ligament (ACL) enables stable knee kinematics by limiting tibial rotation and anterior tibial translation. ACL reconstruction (ACLR) is performed to improve knee stability and shows overall satisfactory results and low revision rates. 1 2 Numerous studies have investigated factors which are associated with the etiology of ACL injury and ACLR failure to reduce ACLR failure rates. 3 4 5 6 7 In addition to neuromuscular control and other physiological factors, the geometrical shape of the knee joint including the posterior tibial slope and the meniscal–cartilage interface have been shown to affect the risk of ACL injury. 8 9 10 However, the influential strength of these geometrical factors and the influence on the risk of ACL failure are not yet fully understood. Christensen et al found that the tibial posterior slope is increased in patients with early graft failure after ACLR; a six-degree increase of tibial cartilage slope resulted in a 10 times higher risk of ACL graft failure. 11 Sturnick et al found that reduced meniscal bone angle (MBA) is associated with increased risk of ACL injury in females. 10 These findings could support the concept that increased tibial slope increases ACL graft strain, while a functional lateral meniscus contributes to restrain against tibial anterior translation and rotation and consequently may reduce graft strain. 12 13 14 15 16 17 Consequently, the integrity and geometrical shape of the lateral meniscus are of paramount importance for knee stability and may affect ACLR outcome. 18 The susceptibility of ACL graft failure is associated with numerous dependent and independent geometrical factors including notch width, meniscal slope, meniscal height, cartilage slope, and MBA. 10 Lateral tibial posterior slope (LTPS) and MBA represent two independent geometrical factors which may affect the risk of ACL failure and which may eventually potentiate or neutralize each other. LTPS and MBA can be assessed on magnetic resonance imaging (MRI) on the same slide with high reliability. 10 19 The purpose of this study was to investigate whether reduced MBA is associated with increased risk of ACL graft failure and if the LTPS–MBA ratio represents a feasible method for the assessment of ACLR failure risk.

## Material and Methods

**Fig. 1:**
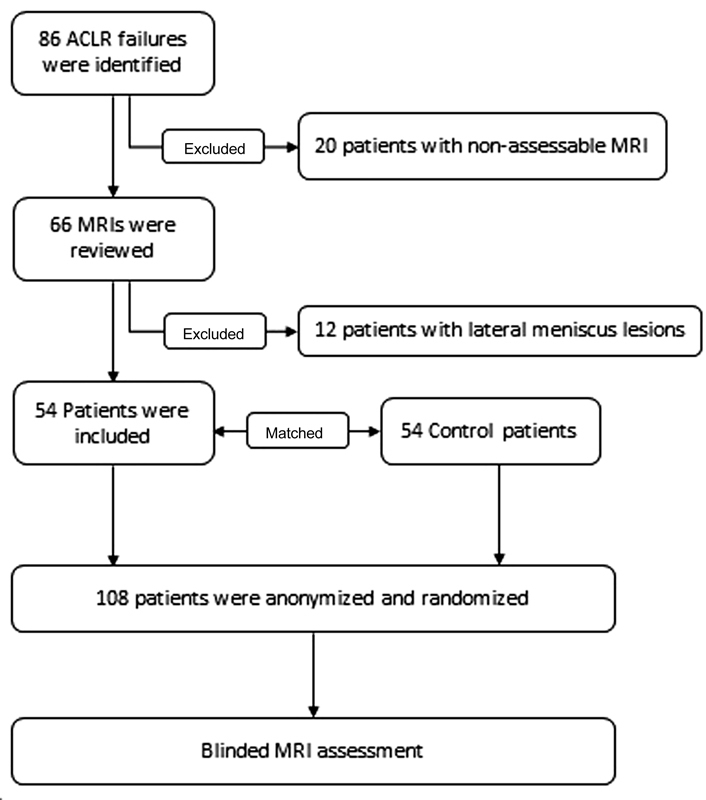
Flowchart describing the criteria used for selection of patients included in this study.

**Fig. 2:**
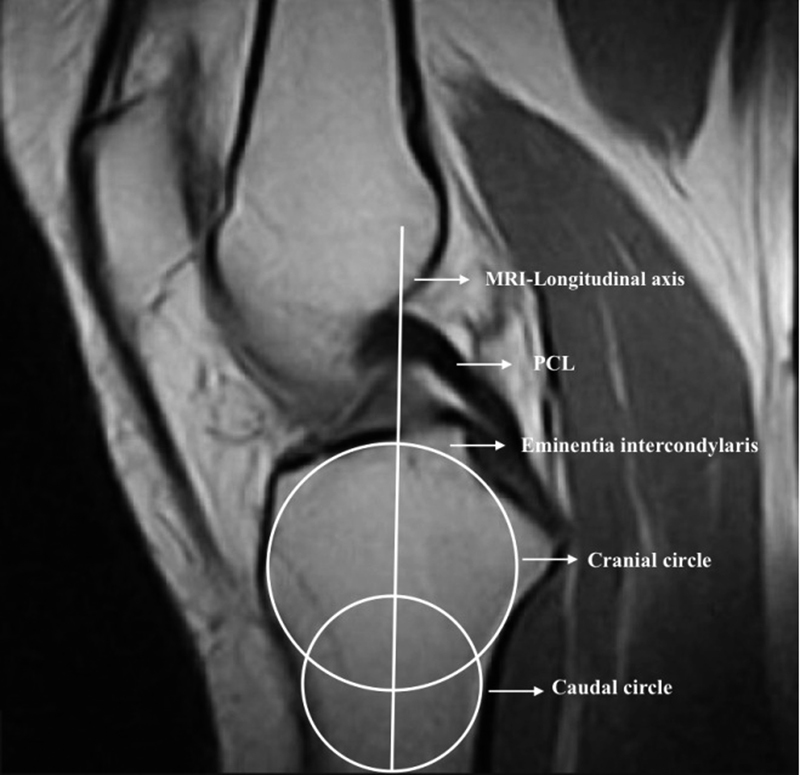
The measuring technique described by Hudek et al. In the first step, the central sagittal image is identified in which the tibial attachment of the posterior cruciate ligament (PCL) and the intercondylar eminence is seen. Subsequently, two circles are placed in the tibial head. A cranial circle which touches the anterior, posterior, and cranial cortex and a caudal circle which touches the anterior and posterior cortex. The center of the caudal circle is hereby positioned on the circumference of the cranial circle. The line connecting the centers of both circles is defined as the magnetic resonance imaging (MRI) longitudinal axis of the tibia and is propagated through the sagittal MRI series. The anatomical center of the lateral tibial plateau is identified on axial slides.

**Fig. 3:**
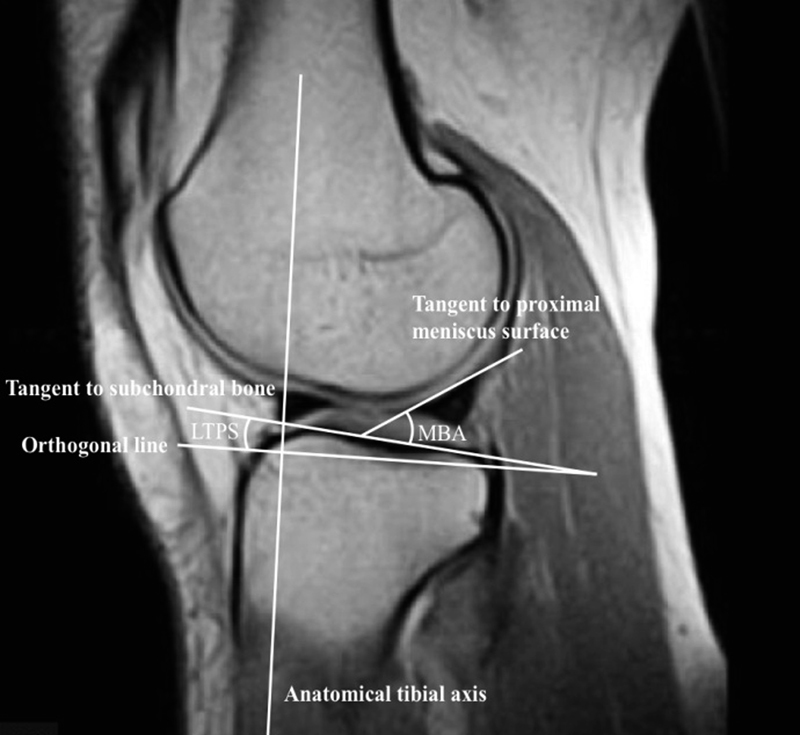
A tangent to the lateral plateau is drawn which connects the uppermost even part between the superior–anterior and posterior cortices. The angle between the orthogonal line to the magnetic resonance imaging (MRI) longitudinal axis and the tangent to the lateral plateau is defined as the lateral tibial posterior slope. The meniscal bone angle is measured as described by Sturnick et al 10 between a tangent to the superior meniscal surface and the tangent to the subchondral tibial bone on the same slide.

After approval by the local ethics committee, a cohort of 1,480 consecutive patients who underwent ACL hamstring reconstruction surgery by a single surgeon between August 2000 and May 2013 was reviewed. In the hospital database, 86 ACLRs had been identified as failures by means of clinical failure and MRI with or without subsequent ACL revision surgery. Clinical failure was defined as ACL graft rupture or insufficiency with consecutive subjective instability and abnormal laxity upon clinical examination. Laxity was not consequently quantified by means of an arthrometer. All patients were followed prospectively with active evaluation 1 year postoperatively and hereafter by need-based appointments. Patients were hereby advised to contact the clinic in case of complications including recurrent instability with or without preceding relevant trauma. Hamstring grafts, suspensory femoral fixation, and a tibial interference screw were used in all cases. Exclusion criteria ( Fig. 1 ) comprised nonaccessible MRI (20 patients) and the presence of MRI-verified lateral meniscus lesions as these lesions could theoretically alter the MBA (12 patients). Fifty-four patients (32 males; 22 females) with ACL graft failure were finally included in this study. Twenty-eight patients had undergone transportal ACLR, while 26 patients had undergone transtibial ACLR. Patients were matched 1:1 by age, sex, graft, fixation method, and surgical technique with 54 control participants, who had undergone ACLR with a minimum of 4 years of follow-up without signs of graft failure. Patients were hereby actively evaluated 1 year postoperatively and hereafter advised to contact the clinic in case of complications including recurrent instability with or without preceding trauma. A total of 108 patients were anonymized and randomized for blinded assessment. MRI (minimum 1.5 Tesla) was used to determine the lateral tibial slope based on the technique described by Hudek et al. 9 The first step of this technique consists in finding the central sagittal image in which the tibial attachment of the posterior cruciate ligament and the intercondylar eminence is seen ( Fig. 2 ). Subsequently, two circles are placed in the tibial head. A cranial circle which touches the anterior, posterior, and cranial cortex and a caudal circle which touches the anterior and posterior cortex. The center of the caudal circle is hereby positioned on the circumference of the cranial circle. The line connecting the centers of both circles is defined as the MRI longitudinal axis of the tibia and is propagated through the sagittal MRI series. In the following step, the axial anatomical center of the lateral plateau is identified and a tangent to the lateral plateau is drawn which connects the uppermost even part between the superior–anterior and posterior cortices. The angle between the orthogonal line to the MRI longitudinal axis and the tangent to the lateral plateau is defined as the LTPS. The MBA was measured as described by Sturnick et al 10 between a tangent to the superior meniscal surface and the tangent to the subchondral tibial bone on the same slide (see Fig. 3 ). The measuring method described by Hudek et al 9 has previously been validated showing excellent reliability (Typical Error [TE] ± 1.4° for interobserver reproducibility and ± 1.2° for intraobserver reproducibility; Correlation Coefficiant [CC] 0.80 for intraobserver and 0.77 for interobserver reproducibility). The measuring technique described by Sturnick et al 10 has previously been validated showing excellent reliability (intraclass correlation coefficient intraobserver 0.9). 20 LTPS and MBA were assessed on MRI after ACL injury and before primary ACLR. All measurements were conducted by a single blinded observer on a radiology suite computer with the necessary software (OsiriX). Data are presented as mean values ± standard deviation and were investigated using logistic regression and receiver operating characteristic curve estimation by an independent professional statistician. For all analyses, a *p* -value of < 0.05 was considered significant.

## Results

In this cohort, 39 patients (36%) showed a LTPS–MBA ratio of under 0.27 which was associated with a 28% risk of ACL failure, while 33 patients (31%) showed a ratio exceeding 0.42 which was associated with an 82% risk of ACL failure. Odds of ACL failure increased by 22.3% per degree of decreasing MBA (odds ratio [OR], 1.22; 95% limits, 1.1–1.34). The ACLR failure group showed a significantly reduced mean MBA of 20.5° ± 3.9° (range, 12.7°–28.7°) compared with the control group with 24.5° ± 4.6° (16.3°–32.6°; *p* < 0.001). Regarding the entire study population, no significant association was found between LTPS and the risk of ACL graft failure (OR, 1.11; 95% limits, 0.96–1.29). In the transportal ACL failure group, the odds of ACL failure increased by 34.9% per degree of increasing LTPS (OR, 1.34; 95% limits, 1.01–1.79), while no significant correlation was found between LTPS and the risk of graft failure in the transtibial ACL failure group. The results are summarized in Tables 1 2 3 . The entire ACL failure group including transportal and transtibial ACLR failures showed an increased LTPS of 7.9° ± 2.8° (range, 2.2–15.5) compared with the control group with 7.1° ± 2.8° (range, 3.2°–16°; *p* = 0.15), which was not significant. When investigating subgroups, the transportal ACLR failure group showed a significantly increased mean LTPS of 8.58° compared with the control group with 7.16° ( *p* = 0.028), see Table 4 . In the isolated transportal ACLR group ( *n* = 28), a LTPS–MBA ratio of under 0.27 was associated with a 12% risk of ACL failure (34% of patients), while a LTPS–MBA ratio exceeding 0.47 was associated with a 98% risk of ACL failure (29% of patients). LTPS and MBA of the transportal and transtibial ACLR groups are presented in Table 5 . No significant correlation was found between MBA and LTPS ( *p* = 0.5).

**Table 1:** LTPS and MBA values of the ACL failure group and matched control group

		LTPS	MBA
Controls ( *n* = 54)	Mean ± SD (min–max)	7.13° ± 2.43° (3.2°–16°)	24.5° ± 4.62° (16.3°–32.6°)
Failures ( *n* = 54)	Mean ± SD (min–max)	7.86° ± 2.81° (2.2°–15.5°)	20.53° ± 4.14°(12.7°–28.7°)
*p* -Value	Differences, means	0.15	< 0.001
Odds ratio	Risk of failure	1.11 (11.4% per > unit)	1.22 (22.3% per < unit)

Abbreviations: ACL, anterior cruciate ligament; LTPS, lateral tibial posterior slope; MBA, meniscal bone angle; SD, standard deviation.

**Table 2:** Results for a LTPS–MBA ratio cut-point of 0.27

	Controls	Failures		
≤ 0.27 ( *n* = 39/108)	28	11	Sensitivity	80%
> 0.27 ( *n* = 69/108)	26	43	Specificity	52%
*N*	54	54	Negative predictive value	72%
			Positive predictive value	62%

Abbreviations: LTPS, lateral tibial posterior slope; MBA, meniscal bone angle.

**Table 3:** Results for a LTPS–MBA ratio cut-point of 0.42

	Controls	Failures		
≤0.42 ( *n* = 75/108)	48	27	Sensitivity	50%
> 0.42 ( *n* = 33/108)	6	27	Specificity	89%
*N*	54	54	Negative predictive value	64%
			Positive predictive value	82%

Abbreviations: LTPS, lateral tibial posterior slope; MBA, meniscal bone angle.

**Table 4:** LTPS of the transportal and transtibial ACLR failure group and control group

	LTPS failures	LTPS controls	*p* -Value
Transportal ACLR ( *n* = 26)	8.58	7.16	=.028
Transtibial ACLR ( *n* = 28)	7.2	7.1	=.9

Abbreviations: ACLR, anterior cruciate ligament reconstruction; LTPS, lateral tibial posterior slope.

**Table 5:** MBA of the transportal and transtibial ACLR failure group and control group

	MBA failures	MBA controls	*p* -Value
Transportal ACLR ( *n* = 26)	25.0	20.7	<0.001
Transtibial ACLR ( *n* = 28)	24.0	20.3	0.003

Abbreviations: ACLR, anterior cruciate ligament reconstruction; MBA, meniscal bone angle.

## Discussion

The primary finding of this study was that reduced MBA is associated with increased risk of ACL graft failure, regardless of ACLR technique and graft positioning. Second, increased LTPS was associated with significantly increased risk of ACL graft failure in the transportal failure group, while no significant association was found in the transtibial failure group. The results of this study suggest that the tibial slope has a higher impact on transportal ACLR compared with transtibial ACLR failure risk, while the MBA effects transportal and transtibial ACLR similarly; the reasoning for this discrepancy remains unknown and needs to be further investigated. However, a possible explanation could be that slope-related graft strain may be potentiated by nonisometric graft positioning, as in transportal ACLR, where the femoral tunnel had been placed central in the ACL footprint. When examining the entire ACL failure group, 36% of the patients showed a LTPS–MBA ratio of under 0.27 and this was associated with a 28% risk of ACL failure, while 31% of the patients showed a ratio exceeding 0.42 which was associated with an 82% risk of ACL failure. The given cut-off points were derived from a logistic model for feasible sensitivity and specificity as well as negative and positive predictive values. No correlation between MBA and LTPS was found.

**Fig. 4:**
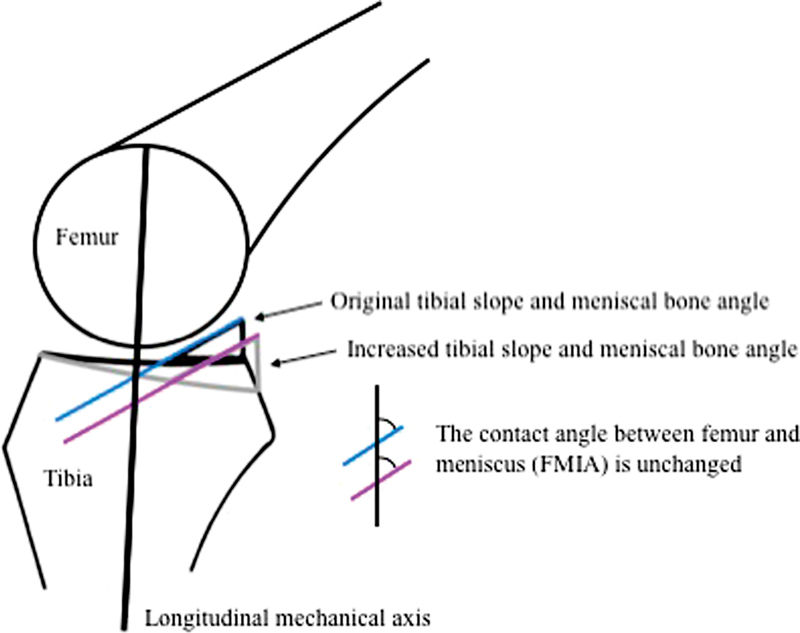
To maintain the same functional femoral meniscal interface angle (FMIA), the meniscal bone angle (MBA) needs to increase if lateral tibial posterior slope (LTPS) increases.

**Fig. 5:**
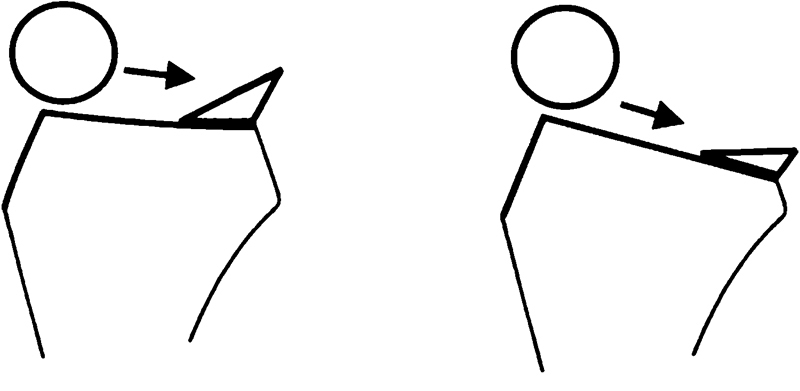
( **A** ) Increased meniscal bone angle (MBA) and reduced lateral tibial posterior slope (LTPS) compared with ( **B** ), resulting in a reduced femoral meniscal interface angle (FMIA). The reduced FMIA may be more effective for deceleration of the femur (circle) in pivoting maneuvers. In conjunction with active muscular stabilization, the meniscus may impede tibiofemoral subluxation and subsequent anterior cruciate ligament (ACL) injury. Increased LTPS may accelerate the femur in pivoting maneuvers and may be best counteracted by a meniscus with increased MBA.

Recent studies have underlined the contribution of the lateral meniscus to rotational knee stabilization, especially in ACL deficient knees. 8 18 21 22 Getgood et al suggested that the lateral meniscus should be regarded as a part of the anterolateral capsulomeniscal complex stabilizing rotation in conjunction with the ACL. 8 Interestingly, Sturnick et al found that decreased MBA is associated with primary ACL injury in females, 10 which supports the assumption that the geometrical shape of the lateral meniscus affects ACL strain forces in normal knees. Furthermore, the geometrical shape of the lateral meniscus may become more influential in ACL reconstructed knees where rotational stability is not fully restored. This may not only have implications for meniscal treatment procedures but also for the assessment of ACL failure risk depending of geometrical factors of the knee joint. Recent studies have shown that increased tibial slope is associated with increased risk of ACL injury and ACLR failure. 17 19 23 Increased anterior tibial translation is thought to be the primary mechanism for this finding. 19 As the position of the menisci is dependent on the underlying surface, it seems conceivable that increased LTPS levels out the femoral meniscal interface angle (FMIA) without substantially affecting MBA, while increased MBA steepens the FMIA without affecting LTPS ( Fig. 4 ). This could be the explanation why increased MBA may theoretically neutralize increased LTPS ( Fig. 4 ) and why the combination of increased LTPS and decreased MBA may be associated with increased risk of graft failure ( Fig. 5 ). ACL rupture results in subluxation of the tibiofemoral joint. Increased lateral tibial slope may increase the acceleration of this event, which normally is counteracted by the posterior meniscal horn ( Fig. 5 ). A dysfunctional posterior meniscal horn may not decelerate this event sufficiently resulting in higher ACL strain and eventually ACL rupture. Increased MBA supports the deceleration of the pivoting event and may therefor reduce the likelihood of ACL rupture. Further studies are needed including weight-bearing MRI to investigate the effect of axial loading on the FMIA. In addition, it remains unclear, to what extend passive stabilization as by the menisci is accountable for deceleration of the femur in pivoting events in contrast to active muscle stabilization, which may be of greater importance.

However, the susceptibility of ACL injury and ACLR failure is multifactorial including the surgical technique, neuromuscular conditions, patient age, level of function, timing of return to sport, compliance to rehabilitation protocols, acquired concomitant injuries, and structural anatomy of the knee joint. 3 4 5 6 7 23 24 25 26 27 28 29 Some factors may also be mutually dependent, e.g., young patient age has been shown to be associated with increased tibial cartilage slope which itself is associated with higher incidence of concomitant meniscal injury and early graft failure. 11 An increasing body of literature is indicating that the cartilage and meniscal slopes may play a more important role for knee kinematics than subchondral slopes. Christensen et al found that the tibial posterior cartilage slope is increased in patients with early graft failure after cruciate ligament reconstruction, 11 while Sturnick et al found that reduced MBA is associated with increased risk of ACL injury in females. 10 In addition, an association between meniscal slope and increased risk of ACL injury has been described. 30 However, the reliability of some measuring techniques has been questioned. Meniscal slope is measured by connecting the peaks of the anterior and posterior horn of the meniscus and it seems conceivable that the geometry of the posterior meniscal horn may have been the primary mechanism for this previously described association. 10 Furthermore, cartilage slope is measured as a tangent to an eventually convex cartilaginous center of the lateral tibiofemoral compartment, which may impede measurement accuracy and reproducibility. Sturnick et al depicted statistically significant relationships between several geometrical intra-articular features including a correlation between MBA and the meniscus–cartilage height. 10 The assessment of these geometrical features using MRI is controversially discussed. Even though high measures of reliability for several slope measurement methods are reported in the literature, 9 10 there is disagreement regarding the actual slope values 19 ; key slope cut-off points which are associated with significantly increased risk of ACL failures are therefore difficult to determine. In this study, we have focused on two geometrical features without substantial correlation that may affect graft strain and which can be measured on the same MRI slide with high reliability. 10 19 Furthermore, the ratio of LTPS and MBA may be of greater importance than the actual slope value, as it might be conceivable that MBA may neutralize or potentiate LTPS and vice versa.

An increasing body of research is emphasizing the contribution of the lateral meniscus to sagittal knee stability 18 22 and the importance of meniscal integrity for better ACLR outcomes. Consequently, meniscal repair procedures including the transtibial technique for meniscal root repairs have been popularized. 21 However, the exact contribution of the lateral meniscus depending on the geometrical shape and integrity is not yet fully understood. Cho et al found that a simple tear of the lateral meniscus does not increase localized pressure in porcine knees when the meniscofemoral ligament is intact 31 ; this may explain why these tears are rarely symptomatic in human knees. 11 Future studies are needed to deepen the knowledge regarding the function of the lateral meniscus and meniscofemoral ligaments as well as the role of suturing techniques for the maintenance of the MBA. The results of this study support the concept that the lateral meniscus has an important role regarding knee stabilization and that the integrity and geometrical shape may affect ACLR outcomes.

The results of this study emphasize that geometrical features of the knee joint including tibial slope as well as the cartilage–meniscal interface may affect the risk of ACL failure. It is not intended to reveal actual LTPS and MBA cut-off points for clinical practice. Slope correcting osteotomies should be reserved for special cases. 32 33 However, a standardized method for assessment of LTPS and MBA may be useful for the assessment of ACL failure risk and may have implications for graft choice or the use of extra-articular stabilizing procedures. This study has limitations. Patients were actively evaluated 1 year postoperatively and hereafter advised to contact the clinic in case of complications including recurrent instability with or without preceding trauma. The true number of failures beyond the first postoperative year is unknown and probably underestimated, as not all patients with graft failure are assessed. It is conceivable that patients with reduced tibial slope and graft failure might refrain from reassessment as they do not experience substantial instability. This could represent a potential bias. Other limitations include the small number of patients and the risk of confounding as other factors may influence the results of this study including patient age, sex, activity level, other geometrical features of the knee as cartilage slope and height, the condition of meniscal tissue, physiological factors such as neuromuscular control and quadriceps-dominant deceleration, as well as hormonal factors. MBAs have been measured on conventional MRI without axial loading of the lower limb which theoretically could alter the MBA. Further studies are needed to evaluate if preoperative assessment of ACLR failure risk based on the geometrical shape of the knee joint is a useful procedure.

## Perspective

A growing body of research is indicating that the tibial slope and the geometry of the tibiofemoral meniscal–cartilage interface affect the risk of ACL injury. 10 11 Increased tibial slope (LTPS) may accelerate pivoting kinematics while the menisci may be of paramount importance for deceleration of these events. To our knowledge, this is the first study to combine the tibial slope and MBA to assess the risk of ACL failure. In the future, MRI-based assessment of geometrical features of the knee joint prior to ACLR surgery may help to identify patients at high risk of ACLR failure. This may have implications for patient counseling and the indication of additional extra-articular stabilizing procedures.
